# Isolation and Expression Analysis of STAT Members from *Synechogobius hasta* and Their Roles in Leptin Affecting Lipid Metabolism

**DOI:** 10.3390/ijms17030406

**Published:** 2016-03-22

**Authors:** Kun Wu, Xiao-Ying Tan, Chuan-Chuan Wei, Wen-Jing You, Mei-Qin Zhuo, Yu-Feng Song

**Affiliations:** 1 Key Laboratory of Freshwater Animal Breeding, Ministry of Agriculture of P.R.C., Fishery College, Huazhong Agricultural University, Wuhan 430070, China; wk901012@126.com (K.W.); weicc993793524@126.com (C.-C.W.); ywj0861@126.com (W.-J.Y.); zhuomeiqin1001@foxmail.com (M.-Q.Z.); maomao0123456@126.com (Y.-F.S.); 2 Collaborative Innovation Center for Efficient and Health Production of Fisheries in Hunan Province, Changde 415000, China

**Keywords:** STATs, gene cloning, molecular characterization, leptin, lipid metabolism, *Synechogobius hasta*

## Abstract

Signal transducers and activators of transcription proteins (STATs) act as important mediators in multiple biological processes induced by a large number of cytokines. In the present study, full-length cDNA sequences of seven *STAT* members, including some splicing variants different from those in mammals, were obtained from *Synechogobius hasta*. The phylogenetic analysis revealed that the seven *STAT* members were derived from paralogous genes that might have arisen by whole genome duplication (WGD) events during vertebrate evolution. All of these members share similar domain structure compared with those of mammals, and were widely expressed across the tested tissues (brain, gill, heart, intestine, liver, muscle and spleen), but at variable levels. Incubation *in vitro* of recombinant human leptin changed the intracellular triglyceride (TG) content and mRNA levels of several STATs members, as well as expressions and activities of genes involved in lipid metabolism. Furthermore, Tyrphostin B42 (AG490), a specific inhibitor of the Janus Kinase 2(JAK2)-STAT pathway, partially reversed leptin-induced change on STAT3 and its two spliced isoforms expression, as well as expressions and activities of genes involved in lipid metabolism. As a consequence, the decrease of TG content was also reversed. Thus, our study suggests that STAT3 is the requisite for the leptin signal and the activation of the STAT3 member may account for the leptin-induced changes in lipid metabolism in *S. hasta*.

## 1. Introduction

Signal transducers and activators of transcription proteins (STATs) are a family of latent cytoplasmic transcription factors that are activated to participate in gene regulation. There are seven distinct STATs in mammals (STAT1, STAT2, STAT3, STAT4, STAT5A, STAT5B and STAT6) with several conserved domain structures, including an N-terminal domain responsible for dimer–dimer interactions, a coiled coil domain involved in protein–protein interactions, a DNA binding domain, a linker domain, an SRC2 homology (SH2) domain and a transcription activation domain (TAD) [1]. During the past years, STATs have been extensively discussed at the molecular level in mammals. However, limited information is available on STATs in fish. To our knowledge, full-length cDNA sequence of *STAT* repertoires have been determined in mandarin fish, *Siniperca chuatsi* [2], and yellow catfish, *Pelteobagrus fulvidraco* [3], although certain members have been obtained in other fishes [4,5,6,7,8]. In addition, due to the whole genome duplication (WGD) event of the modern bony fish [9], additional *STAT* members may have arisen in teleost. Duplicate *STAT5* genes are also found in both mammals and teleostean fish, but not in birds and amphibians [10]. On the other hand, different alternative splicing leads to the production of some additional proteins, such as the truncated β-isoform, which have been identified for STATs (STAT1, STAT3, STAT4, STAT5a and STAT5b) in mammals [11]. However, the detail of alternative splicing is not clear in fish. Accordingly, identification of STATs genes in other teleost species is a key step for characterizing the structure, function and evolutionary history of STATs.

In response to a large number of extracellular signaling polypeptides, STATs are activated by receptor-associated kinase and then transduce signals to the nucleus for transcription regulation. Leptin, as a member of the class-1 cytokines, is considered to exert its biological actions primarily through the Janus Kinase (JAK)-STAT signaling pathway in mammals. In fish, the physiological effects of leptin on energy metabolism have been well characterized [12,13,14,15,16]. Recently, by using a specific pathway inhibitor, subdued effects of leptin were observed in hepatocytes of grass carp, *Ctenopharyngodon idellus* [15], and yellow catfish, *Pelteobagrus fulvidraco* [16], which have provided us some important, yet indirect evidence about the close relationship between leptin’s role in lipid metabolism and the JAK-STAT signaling pathway. However, due to the absence of individual sequence information of *STATs*, mRNA expression of *STATs* members was absent in those studies and the manner by which STAT member leptin directly affected lipid metabolism was not well understood. In addition, since multiple isoforms arose from the teleost-specific WGD event and different transcript variants may play distinct physiological roles, it seems absolutely necessary to study whether different STAT members respond differentially to the leptin signaling.

*Synechogobius hasta* (Perciformes: Gobiidae), a carnivorous fish, is considered to be a potential commercial species in northern China because of its euryhalinity, rapid growth and delicious meat [17]. In the present study, full-length cDNA sequences of all *STAT* members were cloned from *S. hasta* and their evolutionary relationships and tissue expression profiles were assayed. Meanwhile, we also determined the role of STATs in leptin influencing lipid metabolism. The present study aims to extend our understanding and provide more details about molecular characterization, physiological function and interaction mechanism of STAT members with leptin in fish.

## 2. Results

### 2.1. Complete Sequences and Phylogenetic Analysis of Signal Transducers and Activators of Transcription (STATs)

The complete cDNA of the *S. hasta STAT* genes were compiled by overlapping the sequences of the cloned cDNA and the 5′-rapid amplication of cDNA ends (RACE) and 3′-RACE polymerase chain reaction (PCR) products. The *STAT* family consists of seven members: *STAT1*, *STAT2*, *STAT3*, *STAT4*, *STAT5.1*, *STAT5.2* and *STAT6*. The full-length cDNA of *STATs* ranged from 2804 to 3722 bp, with ORFs of 2229–2484 bp encoding 742–827 residues (Table 1). Alternative splicing of STAT1 resulted in the generation of two isoforms, named *STAT1-1* and *STAT1-2*. *STAT3* transcript could be alternatively spliced to generate *STAT3-2*, which lacked the 20 amino acids in TAD compared with STAT3-1.

**Figure 1:**
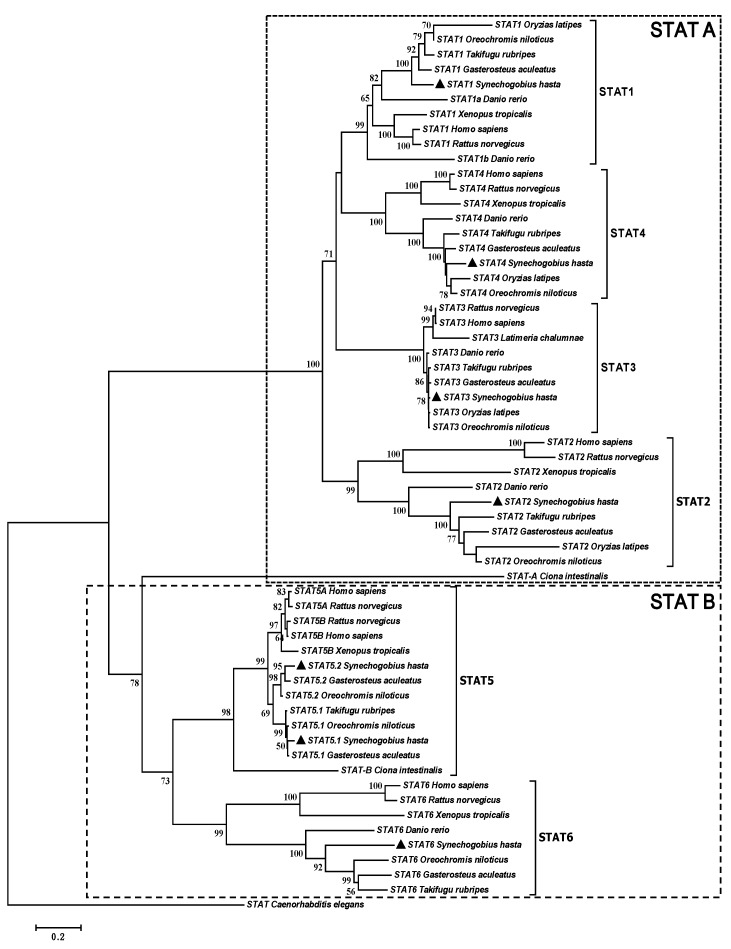
Neighbour-joining phylogenetic tree based on the protein sequences of signal transducers and activators of transcription proteins (STATs) from *S. hasta* (▲) and other species. Branch support values represent a percentage of 1000 bootstrap replicates and values lower than 50 were absent in the present analysis. Protein accession numbers are listed in Supplementary Table S1.

Phylogenetic analysis based on putative amino acid sequences was used to investigate the inferred evolutionary position and relationship of STAT family members among major vertebrates. As shown in Figure 1, two groups are separated with a high bootstrap value, one including STAT1, STAT2, STAT3 and STAT4 and the other including STAT5 and STAT6. In each STAT clade, *S. hasta* clustered with other teleosts to form a distinct subclade, but separated from amphibians and mammals. On the basis of the tree topologies, there appeared to be a gene duplication resulting in two STAT5 members in both fish (STAT5.1/2) and mammals (STAT5 A/B). Moreover, STAT5 displayed a higher sequence conservation than other STATs, as it showed shorter branch lengths.

### 2.2. STATs Structure and Sequence Analysis

**Figure 2:**
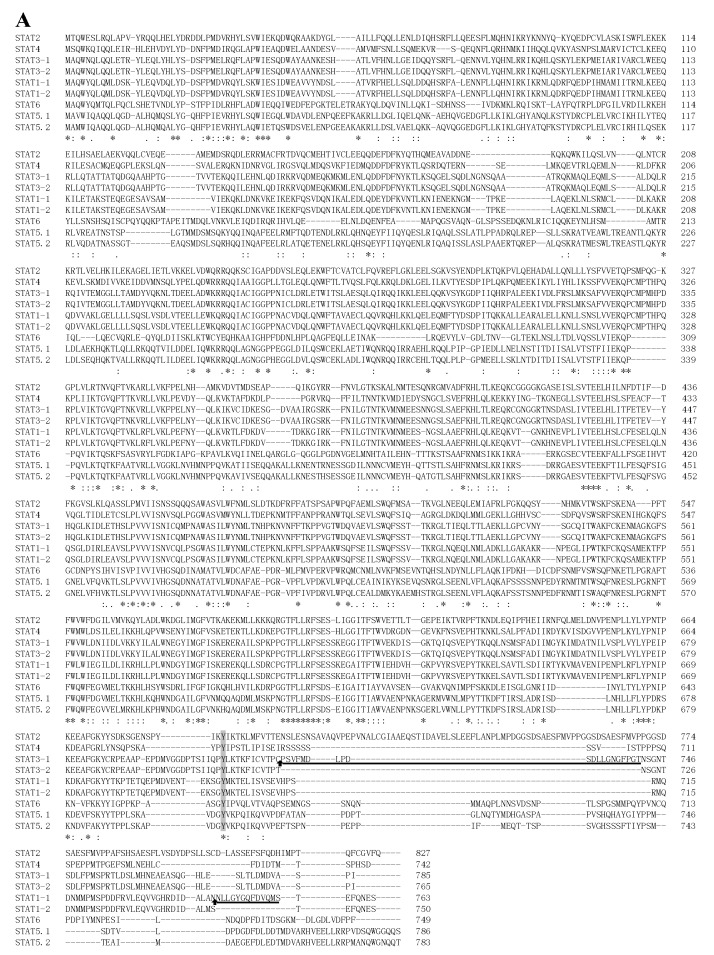

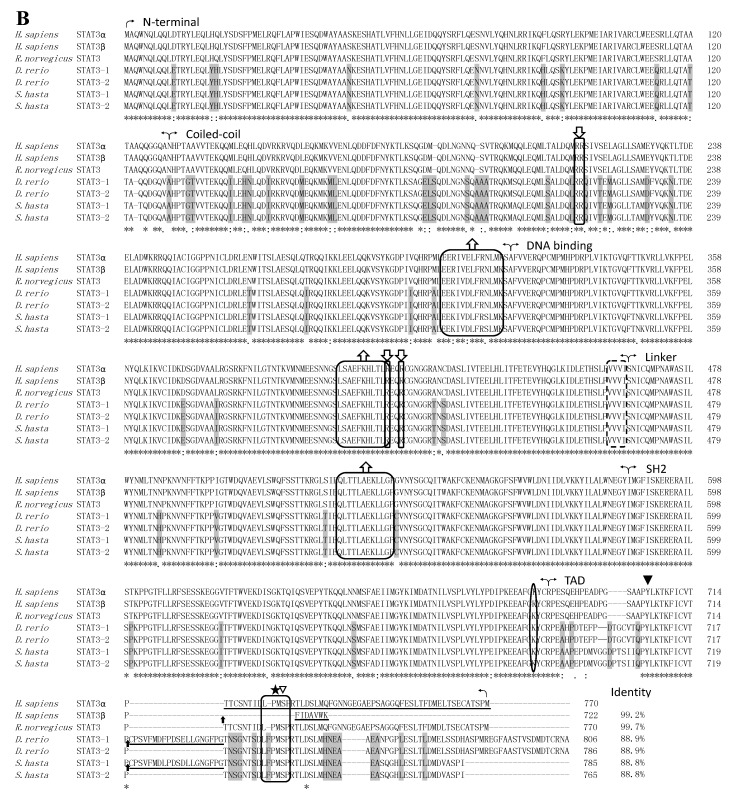

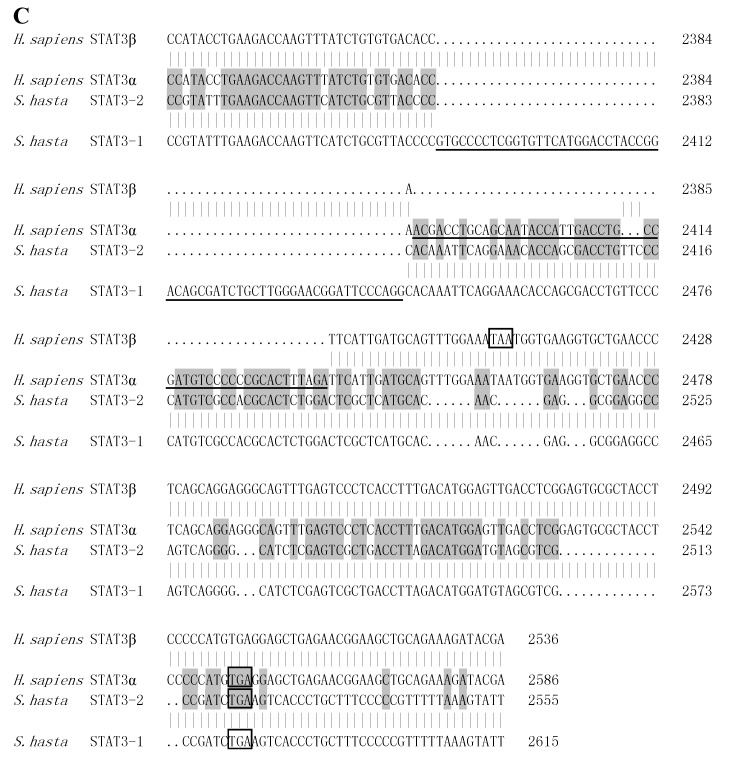
Multiple sequence alignment of STATs. The accession numbers from Ensemble databases are given in Supplementary Table S1. Numbers on the right indicate the amino acid positions. Identical (*), high similar (:) and low similar (.) residues were indicated below the alignment. Hyphens represent spaces inserted to maximize similarity. (**A**) Alignment of the deduced amino acid sequences of STATs from *S. hasta*. Spliced sites are indicated by upward arrowheads. Conserved phosphorylation sites in all STAT members are shaded by a dark background. Different amino acids generated by alternative splicing are indicated by an underline; (**B**) Alignment of STAT3 amino acid sequences from *S. hasta*, *D. rerio*, *H. sapiens* and *R. norvegicus*. Curved arrowheads represent the boundaries of different domains. Upward arrowheads represent spliced sites. Different amino acids generated by alternative splicing are indicated by an underline. A solid triangle represents a single phosphotyrosine that is present in all activated STATs, and an open triangle represents a single phosphoserine that is present in several STATs [1]. A single lysine residue that regulates STAT3 dimerization by reversible acetylation is denoted by an oval frame. Boxes and stars represent the LPMSP motif [18]. Nuclear export signals (NES) are indicated by a box and an upward arrowhead [19]. Nuclear localization signals (NLS) are indicated by a box and a downward arrowhead. A dotted box represents the region that plays a role in nuclear translocation induced by growth hormone in STAT5B, but mutation of it has no effect in STAT3 [20]. The amino acid divergence between fish and mammals is shaded by a dark background; (**C**) Alignment of STAT3 nucleotides sequences from *S. hasta* and *H. sapiens*. The conserved nucleotides of STAT3 in the same species are indicated by a vertical line. The conserved nucleotides between *H. sapiens* STAT3α and *H. hasta* STAT3-2 are shaded by a dark background. The positions of the stop codons are indicated by a box. Different cDNA sequences generated by alternative splicing are indicated by an underline.

Amino acid sequence identity of STATs between different species are shown in Table 2. Generally speaking, the same isoforms shared the maximal identical residues compared to the different isoforms in different species. STAT3 between different species shared the average maximal identical residues (92.4%), while the minimum identity was found in STAT6 (50.8%). Among different STAT members of *S. hasta*, STAT5.1 and STAT5.2 shared the highest identity (81.4%) while STAT2 and STAT6 showed the lowest identity (22.9%) (Figure 2A). Similar to the ortholog of mammals, *S. hasta* STAT molecules can be divided into six functional domains: an N-domain responsible for dimer–dimer interactions, a coiled coil domain responsible for protein–protein interactions, a DNA-binding domain (DBD), and a linker domain implicated in transcription, an SH2 domain responsible for receptor binding and dimerization and a transcriptional activation domain (TAD). The overall identity between the *S. hasta* STAT3 and other STAT3s was very high (Figure 2B). In mammals, STAT1, STAT3 and STAT4 have a Leu-Pro-Met-Ser-Pro motif containing the active-site serine residue. In the same position as *S. hasta* and zebrafish STAT3, we also found the analogous motif with an additional Phe residue. Three presumable nuclear export signal (NES) structures, EEKIVDLFRSLMK at 307–319 amino acids, LSAEFKHLTLR at 405–415 amino acids and QLTTLAEKLLGP at 525–536 amino acids, were found in the present study. On the other hand, R215–R216, R415 and R418 might be potential residues involved in nuclear translocation. In addition, there were 65 potential fish-specific substitutions, since these residues were identical among fish but different from mammalian sequences.

In addition, we analyzed the details of STAT3 splicing in *S. hasta* and *H. sapiens* (Figure 2C). Due to the deletion in STAT3β, a stop codon is generated in advance and the reading frame is switched. In contrast, in *S. hasta*, 60 nucleotides were inserted, which led to the production of an additional 20 residues in STAT3-1. These results suggested that neither STAT3-1 nor STAT3-2 is analogous to STAT3β.

### 2.3. Expression of S. hasta STATs in Various Tissues

**Figure 3:**
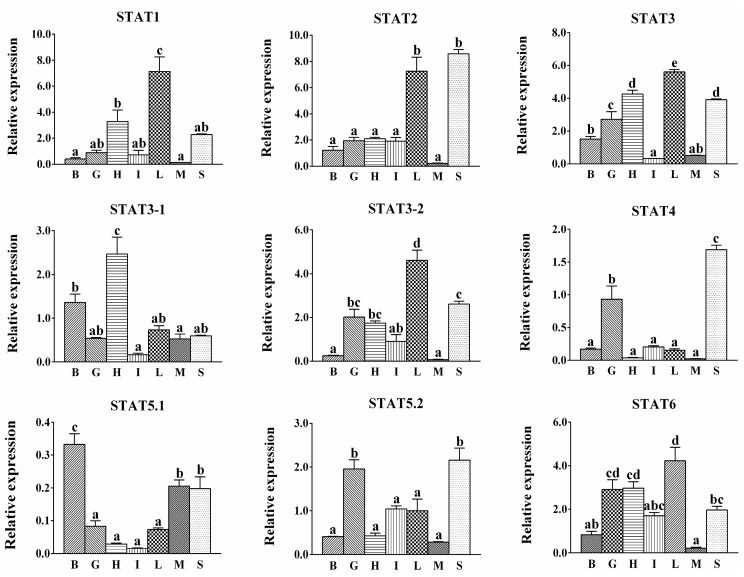
Quantitative PCR (Q-PCR) analysis for expression of *STATs* across brain (B), gill (G), heart (H), intestine (I), liver (L), muscle (M) and spleen (S) of *S. hasta*. Data (mean ± SEM, *n* = 6 specimens, mixed sexes) were expressed relative to expression of housekeeping gene (*β-Actin* and *18 s rRNA*). The expression of *STAT5.2* in liver was arbitrarily set as 1, for calculation of relative expression in other tissues. Bars that do not share the same lowercase letter mean significant difference among different tissues (*p* < 0.05).

In general, all *STAT* members were broadly distributed, but at varying levels with relatively lower expression in muscle (Figure 3). The expression of *STAT1*, *STAT2*, *STAT3* and *STAT6* were at relatively higher levels in most tissues, while *STAT4*, *STAT5.1* and *STAT5.2* were expressed at low levels. For each *STAT* member, *STAT1* was the most abundant in liver, followed by heart, and no significant differences were found among other tested tissues. *STAT2* mRNA levels were the highest in liver and spleen, and at relative lower levels in other tissues. *STAT3* mRNA levels were the highest in liver, the lowest in muscle and intestine. For two spliced isoforms of *STAT3*, in brain, heart and muscle, *STAT3-1* were predominantly expressed. In liver and spleen, the results were the opposite. The highest mRNA levels of *STAT4* were found in spleen, followed by gill, and no significant differences were found in other tested tissues. *STAT5.1* expression level was the highest in brain, followed by muscle and spleen, and the lowest in other tested tissues. *STAT5.2* mRNA levels were the highest in gill and spleen, and no significant differences were found in other tested tissues. *STAT6* mRNA levels were the highest in liver, the lowest in brain and muscle, and showed no significant differences among other tissues.

### 2.4. Cell Viability and Intracellular Triglyceride (TG) Content

**Figure 4:**
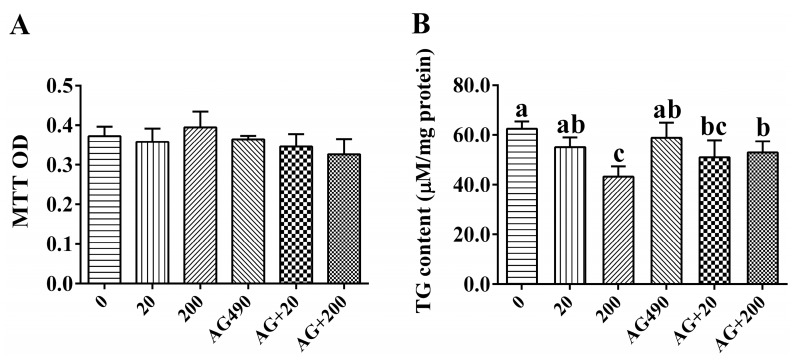
(**A**) Effect of leptin and/or Tyrphostin B42 (AG490) on cell livability in primary hepatocytes from *S. hasta*; (**B**) Effect of leptin and/or AG490 on hepatocytes TG accumulation. Values are mean ± SEM (*n* = 4). Bars that share different letters indicate significant differences among groups (*p* < 0.05), and bars without different lowercase letters indicate no significant differences among groups (*p* < 0.05).

Leptin and the specific inhibitor AG490 of JAK2/STAT pathway had no adverse effect on cell viability during the experiment (Figure 4A). In the *in vitro* experiment, 20 nM leptin had no significant effect on intracellular TG content, but 200 nM leptin significantly reduced the TG content at 48 h. The effect of leptin on TG content was partly suppressed by AG490 (Figure 4B).

### 2.5. Enzymatic Activities

**Figure 5:**
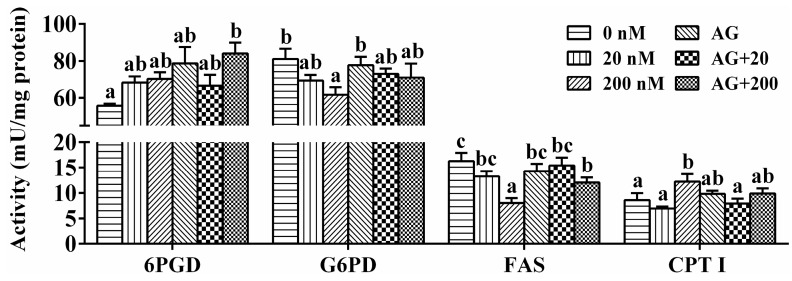
Effect of leptin and/or pathway inhibitor AG490 on enzymatic activities in the hepatocytes of *S. hasta in vitro* at 48 h. Values are expressed as mean± SEM (*n* = 4). Bars that share different lowercase letters indicate significant differences among groups (*p* < 0.05).

As shown in Figure 5, 20 nM leptin incubation showed no significant effects on enzymatic activities. In contrast, 200 nM leptin improved CPT I activity at 48 h. The activities of G6PD and FAS at the 200 nM leptin group were significantly lower than those in the control group. For all the tested enzymes, single AG490 incubation showed no significant effects on their activities. Compared to single leptin treatment, AG490 pretreatment repressed the changes of G6PD, FAS and CPT I activities.

### 2.6. Transcriptional Regulation of the Genes by Leptin in Vitro

**Figure 6:**
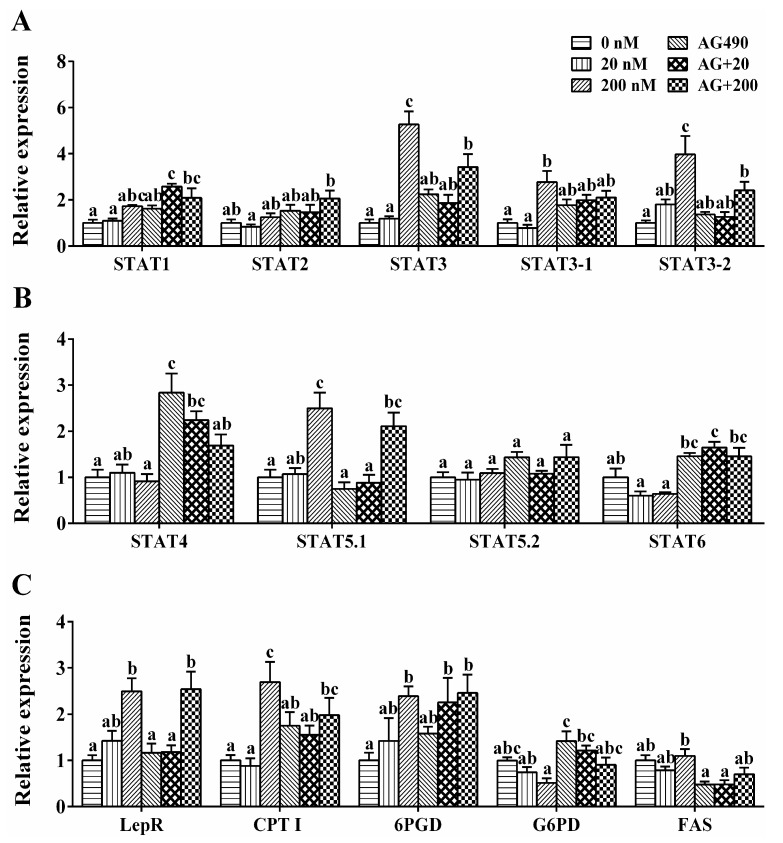
Effects of leptin and/or pathway inhibitor AG490 on the mRNA levels in the hepatocytes of *S. hasta in vitro* at 48 h. (**A**) STAT1-3; (**B**) STAT4-6; and (**C**) LepR and genes involved in lipid metabolism. Data (mean ± SEM, *n* = 4) were expressed relative to expression of housekeeping gene (*RPL7* and *TBP*). Bars that share different lowercase letters indicate significant differences among groups (*p* < 0.05).

In hepatocytes of *S. hasta*, single AG490 showed no significant effect on mRNA levels of *STATs* and enzymatic genes except the mRNA level of *STAT4* (Figure 6). 20 nM leptin incubation had no significant effect on genes’ expression. After 200 nM leptin incubation, mRNA levels of *STAT3*, *STAT3-1*, *STAT3-2* and *STAT5.1* were significantly higher than control groups. *LepR*, *CPT I* and *6PGD* genes expression were up-regulated, while the *G6PD* mRNA level decreased. Compared to single leptin treatment, AG490 pretreatment partly repressed the changes of *STAT3*, *STAT3-1*, *STAT3-2*, *CPT I* and *G6PD* mRNA levels, but showed no significant effect on other genes’ expression.

## 3. Discussion

In the present study, the full-length cDNA sequences of all STAT members (*STAT1, STAT2*, *STAT3*, *STAT4*, *STAT5.1*, *STAT5.2* and *STAT6*), including some splicing variants, were isolated and characterized in *S. hasta*. Furthermore, we investigated their mRNA expression profiles among various tissues and explored the role of STATs and activation of the JAK-STAT pathway in lipid metabolism by leptin stimulation. Since the structure and role of teleost STATs attract little attention at present, our results aim to enhance our understanding on the physiological function of *STAT* genes in lipid metabolism, as well as on the complex signal transduction system in fish.

In this study, at least six *STAT* members were found in *S. hasta*, and clustered with corresponding STATs of other teleost and mammals to form distinct subclades. The topology of our phylogenetic trees suggests that the *S. hasta STAT* family has arisen via a series of duplications of an ancestral gene with a classical WGDs-driven expansion pattern during vertebrate evolution, as suggested in vertebrate [10]. Moreover, we successfully obtained *STAT2* and confirmed the presence of *STAT2* in *S. hasta*, similar to the reports in Atlantic salmon [5] and turbot [8]. On the contrary, Guo *et al.* [2] obtained all *STAT* members from mandarin fish except *STAT2*. Furthermore, two *STAT5* genes exist in *S. hasta*, similar to the study in zebrafish [21]. However, only a single *STAT5* gene has been identified in other teleosts [2,6], suggesting that the presence of two *STAT5* genes may be restricted to limited teleosts. *S. hasta STAT5.2* was separated from the *STAT5.1* of the other teleosts, but clustered with them to form a distinct subclade which diverged prior to the divergence of mammalian *STAT5A* and *STAT5B*. This indicates that they do not represent *STAT5a* or *STAT5b* equivalents. Similarly, Lewis and Ward [21] pointed out that the *SATA5* gene has undergone two independent gene duplication events to generate *STAT5.1*/*STAT5.2* in fish and *STAT5A*/*STAT5B* in mammals. In the phylogenetic tree, all STAT members of *S. hasta* are grouped into two parts with a closer evolutionary relationship. One part contains *STAT1*, *STAT2*, *STAT3* and *STAT4*, and another contains *STAT5.1*/*5.2* and *STAT6*. Similar results were also observed by Guo *et al.* [2] and Wu *et al.* [3].

Multiple sequence alignments revealed that all *S. hasta* STAT proteins share a similar structure, consisting of an N-terminal domain, a coiled coil domain, a DBD, a linker domain, an SH2 domain and a TAD, similar to those in other fish [2,3,4,8]. STATs are a family of latent cytoplasmic proteins that are activated at the plasma membrane by tyrosine phosphorylation and participate in gene control. Up to now, the phosphorylation sites of fish STATs were not determined. In this study, a putative tyrosin phosphorylation site around residue 700 was found in the TAD of all *S. hasta* STATs. Indeed, Tyr phosphorylation is a prerequisite for STAT activation. A conserved tyrosine with approximately 700 residues had been determined in each mammalian STAT protein and its phosphorylation is obligatory for STAT activation [22]. Besides tyrosine, a subset of STAT TADs also use serine phosphorylation to further regulate transcriptional functions. In STAT1, STAT3 and STAT4, there is a highly conserved Ser727 as part of a Leu-Pro-Met-Ser-Pro motif located centrally in the TAD, whose phosphorylation is necessary for maximal activation [18]. The alignment result also reveals that a potential phosphorylation site exists in the above-mentioned motif in the STAT3 of *S. hasta* and zebrafish. However, an additional Phe was inserted in the motif of the two fish, which changed the motif into Leu-Phe-Pro-Met-Ser-Pro, similar to the STAT3 in turbot [7] and yellow catfish [3]. This result may indicate a latent difference in the efficiency of transcriptional regulation between fish and mammals. Like other transcription factors, STATs are able to import/export the nucleus by the nuclear localization signal (NLS)/nuclear export signal (NES) [23,24]. The determined NLSs and NESs of the human STAT3 protein were highly conserved in both *S. hasta* STAT3s, thus the stability of the amino acids may be important for its nuclear translocation. In addition, some amino acids divergence was also observed between fish and mammalian STAT3, indicating the potential different functions in fish and mammals.

In the present study, all STAT members were widely distributed in the tested tissues, but their abundance varied within tissues, similar to those in other fish species [2,3,4,5,7,8]. The ubiquitous expression reflected the importance of STATs in various biological functions, while the difference in tissue-dependent expression probably indicated the tissue-specific regulation in biological function in different tissues. In *S. hasta*, STATs members were expressed at a very low level in muscle, in agreement with the report in mandarin fish [2]. In the present study, *S. hasta* exhibit some differences in the gene expression pattern of *STAT5.1* and *STAT5.2*, in agreement with the study by Wu *et al.* [3]. Given the parallels between these two members, it was plausible that the potential subfunctionalization or diversity of functions exists in them, as suggested by other studies [25,26]. On the other hand, different abundance was observed between two *STAT3* spliced isoforms among various tissues, although the differences multiples were not obvious. The near mRNA levels of them probably indicated that these two isoforms had overlapping functions and exert physiological functions together.

Although considerable studies have described the physiological effects of leptin in fish, the leptin-dependent signal transduction pathway is not well understood. Previous studies in mammals have documented the importance of the JAK-STAT pathway under leptin actions. However, the lack of studies addressing the properties of STATs precludes further speculation in fish. Hence, it is worth investigating which STAT family members play a role in leptin-induced change of lipid metabolism in fish. At the first step, we evaluated whether human leptin has an effect on the lipid metabolism of *S. hasta*. In the present study, 20 nM leptin had no significant effect on mRNA levels of enzymatic genes. However, higher concentration of leptin increased activity and expression of CPT I, but decreased activities of G6PD and FAS. FAS plays an important role in the lipogenic pathways. 6PGD and G6PD are involved in production of NADPH, essential for fatty acid biosynthesis [27]. CPT I is the key enzyme in regulation of mitochondrial β-oxidation because it catalyzes the transfer of acyl-groups into the mitochondrial matrix [28]. Accordingly, the present study indicated that leptin was able to promote lipolysis, probably by down-regulation of the G6PD and FAS activities and up-regulation of *CPT I* at the transcriptional level and enzyme activity level, which supports the lipolytic role of leptin by increasing fat utilization and decreasing fat synthesis *in vitro*. For STAT family members, 200 nM leptin up-regulated the mRNA levels of *STAT3*, including *STAT3-1* and *STAT3-2*, and the mRNA level of *STAT3-2* increased with increasing leptin concentration, indicating that both of the two *STAT3* transcripts responded to leptin stimulation at the transcriptional level. Interestingly, STAT5.1 was also up-regulated after 200 nM leptin incubation at 48 h. Similarly, our recent study also indicated that more than one STAT member’s expressions were up-regulated after leptin incubation in yellow catfish [3]. In fact, as with STAT3, STAT5 is also a member of a STAT subset known as the “fat-STAT” [29]. STATs form homo- or hetero-dimers with other phosphorylated STAT proteins, after which they detach from the receptor and then translocate into the nucleus for gene expression regulation [30]. Thus, it was plausible that more than one STAT member was involved in regulation of lipid metabolism under the leptin signal. Indeed, in COS cells, the STAT5 was activated following ligand binding to the leptin receptor, but the result of STAT6 was equivocal [31].

Further, AG490, a specific and potent inhibitor of the JAK2-STAT pathway, was applied to the incubation. For the key lipid metabolic enzyme, the present study indicated that the effects of leptin on genes expression (*FAS* and *CPT I*) and activity (CPT I) were partly suppressed by AG490. As a consequence, the reduction of hepatocyte TG content by 200 nM leptin stimulation was also partly reverted in the presence of AG490, similar to the studies in other fish [3,15,16]. Compared to single leptin groups, AG490 also significantly reduced mRNA levels of *STAT3*, but showed no significant effects on gene expression of other STAT members. Indeed, STAT proteins are substrates of JAKs and their activation largely depends on JAKs’ phosphorylation [32]. Thus our study suggested that inhibition of activation of JAK2 by AG490 presumably caused the reversion of the leptin-induced effect on *STAT3* expression, which subsequently affected downstream genes’ expression. In addition, the change of two *STAT3* splicing variants’ mRNA levels was also partly reverted after AG490 intervention. Given that both *STAT3-1* and *STAT3-2* possess comparatively complete TAD, the result indicated that two *STAT3* isoforms had overlapping functions and responded to the JAK2 signal together. The leptin receptor is considered to be the connection between the leptin signal and the downstream signaling pathway [33]. However, in the present study, AG490 had no suppressive effect on LepR expressions, indicating that AG490 probably blocked the signal at the step of JAK2 to STAT3, but made no difference to prior steps. In fact, activation of STAT proteins was highly dependent upon the activity of JAK and the number of phosphorylated tyrosines within cytokine receptor/JAK complexes [34]. Thus, taken together with the close correlation of the leptin receptor, JAK2 and STAT3, it was likely that STAT members constitute a pivotal link between the leptin signal and enzymatic actions and that the relevant STAT3 member, at least in part, was involved in the leptin-induced change of lipid metabolism in *S. hasta*.

In this work, seven *STAT* family members, including two splicing variants of *STAT1* and *STAT3*, were isolated and characterized from *S. hasta*. The phylogenetic analysis revealed that the seven STAT members were derived from paralogous genes that might have arisen by WGDs during vertebrate evolution. The widespread tissue expression reflected that distinct tissues were potential STAT targets in various biological roles. Leptin administration *in vitro* reflected the role in regulating the processes of hepatic lipid metabolism presumably via the STATs. Differential expression of relative genes in the presence of AG490 and leptin indicated that the lipolytic action of leptin was mainly exerted through the STAT3 member.

## 4. Materials and Methods

Two experiments were conducted in this study. The first experiment was involved in the *STAT* family members cloning, sequence analysis and mRNA expression patterns of various tissues. The second experiment was designed to investigate the possible roles and mechanism of STAT members in leptin influencing lipid metabolism *in vitro*. We ensured that the experiments performed on animals and cells followed the ethical guidelines of Huazhong Agricultural University, and confirmed that all experimental protocols were approved (HBAC20150310) by Huazhong Agricultural University (date of approval: 10 March 2015).

### 4.1. STAT cDNAs Cloning and mRNA Expression Patterns of Various Tissues

#### 4.1.1. Fish Rearing and Sampling

Juvenile *S. hasta* (body weight: 21.14 ± 2.27 g) were obtained from Panjin Guanghe Fisheries Co. Ltd (Panjin, China). They were maintained in indoor cylindrical fiberglass tanks (300-L water volume) at ambient temperature (25 ± 4 °C) with a natural dark/light cycle for 2 weeks of acclimation. During the acclimation, all the fish were fed twice daily with minced trash fish. Meantime, continuous aeration was provided to maintain high dissolved oxygen concentration in tanks. Water in each tank was renewed by 80% every day. At the end of acclimation, fish were starved for 24 h before sampling. After being euthanized with 3-aminobenzoic acid ethyl ester methanesulfonate (MS-222, 100 mg·L^−1^), liver, brain, gill, heart, white muscle, spleen and anterior intestine were removed on ice, and rapidly frozen in liquid nitrogen and stored at −80 °C for subsequent analysis.

#### 4.1.2. RNA Isolation and cDNAs Synthesis

Frozen tissues were powdered in liquid nitrogen using a mortar and pestle. Total RNA was extracted by the acid guanidinium thiocyanate-phenol-chloroform extraction method as described in the manual of TRIzol RNA reagent (Invitrogen, Carlsbad, CA, USA). The quality of total RNA was assessed by agarose gel electrophoresis. The OD values (OD_260_/OD_280_) of total RNA were determined by a Nanodrop ND-2000 spectrophotometer (Thermo Fisher Scientific, Wilmington, DE, USA). Only those total RNA with OD_260_/OD_280_ value more than 1.8 was used for reverse transcription PCR. Total RNA was reverse transcribed into cDNA using the cDNA Synthesis Kit (TaKaRa, Shiga, Japan).

#### 4.1.3. Cloning and Sequencing of STATs

Different degenerate primers (Supplementary Table S2) were chosen and designed from the conserved area of the fish *STAT* genes for amplification of partial cDNA fragments. The PCR program is as follows: initial denaturation for 4 min at 94 °C, followed by 30 cycles of denaturation for 30 s at 94 °C, annealing at 55 °C for 30 s, extension at 72 °C for 1 min and a final step at 72 °C for 10 min. The PCR products were purified (EZNA gel extraction kit, Omega, Norcross, GA, USA) and then subcloned into the pMD 19-T vector (TaKaRa, Shiga, Japan). The products inserted with the expected fragments were sequenced commercially (Sangon, Shanghai, China). The 3′ and 5′ rapid amplification of cDNA end (RACE) were performed with a commercial kit (SMART RACE cDNA Amplification Kit, Clontech, Mountain View, CA, USA) using the Dnase treated total RNA, with 1 µL 10× Reaction Buffer, 1 U DNase I for 1μg RNA incubated at 37 °C for 15 min, then with 1 µL 25 mM EDTA incubated at 65 °C for 15 min to inactivate the DNase I. The PCR program was conducted at 94 °C for 3 min, followed by 30 cycles of 94 °C for 30 s, 55 °C for 30 s, 72 °C for 1 min, and a final cycle of 72 °C for 10 min. The RACE products were purified, cloned, and sequenced as described above.

#### 4.1.4.Sequence Analysis

The full length *STAT* cDNAs were assembled from sequence fragments using SeqMan II in the DNASTAR package. The STATs amino acids sequences were deduced and analyzed by the program EDINSEQ in the DNASTAR package. The nucleotide sequences were subjected to homology search using the BLAST network service at NCBI. Multiple sequence alignments were performed with the Clustal W algorithm. Domains were analyzed by the SMART program and online CDD tool at NCBI. Putative phosphorylation sites were predicted by KinasePhos and DISPHOS 1.3. For phylogenetic analysis, multiple sequences were aligned using MAFFT [35] and analyzed by the GUIDANCE web-server [36]. MEGA 5.0 [37] was used to construct the phylogenetic tree using the neighbor-joining (NJ) method based on the JTT + G model [38]. The optimal model of evolution was selected by maximum likelihood (ML) model selection. Bootstrap sampling was reiterated 1000 times.

### 4.2. Hepatocytes Treatment in Vitro

#### 4.2.1. Hepatocytes Culture and Treatment

For experiment *in vitro*, hepatocytes were isolated from juvenile *S. hasta* liver according to Liebel *et al.* [39] with slight modification in our laboratory [40]. Cells were counted in a hemocytometer. Trypan blue exclusion was conducted to determine cell viability, and only those cultures with more than 95% cell viability were accepted for the following experiments. The freshly isolated hepatocytes were seeded at a density of 1 × 10^6^ cells/mL onto 25 cm^2^ flasks and kept at 28 °C in a CO_2_ incubator (0.5% of CO_2_). For each cell culture, a pool of cells from four fish was utilized.

Freshly isolated hepatocytes of *S. hasta* were incubated with high-performance liquid chromatography (HPLC)-purified recombinant human leptin (rt-hLEP, Sigma, Carlsbad, CA, USA) and/or AG490 (JAK2-STAT specific inhibitor, Sigma). Here, six groups were designed as follows: control (containing 0.1% DMSO, dimethyl sulfoxide, sigma), leptin (20 ng/mL), leptin (200 ng/mL), AG490 (20 µM), leptin (20 ng/mL) + AG490 (20 µM), leptin (200 ng/mL) + AG490 (20 µM), respectively. Each treatment was performed in quadruplicate. The inhibitor was added 2 h prior to the addition of leptin. The concentration of leptin and specific inhibitor was selected according to our preliminary experiment and to previous *in vitro* studies in fish and mammals [3,14,15,41]. The cells were gathered for the following analysis after 48 h. Lyophilized leptin was dissolved in 1× PBS and AG490 was dissolved in DMSO. The maximal DMSO concentration applied to cells in culture did not exceed 0.1%, and had no major effect on cell viability and parameters.

#### 4.2.2. Cell Viability Assay, TG Content and Enzyme Activity Determination

The 3-(4,5-dimethylthiazol-2-yl)-2,5-diphenyltetrazolium bromide (MTT) assay was used to test the cell viability according to our previous *in vitro* studies [40,42]. Results were presented as the OD value of each treatment group subtracting the positive group OD value. Intracellular triglyceride (TG) content was determined by glycerol-3-phosphate oxidase *p*-aminophenol (GPO-PAP) methods, using a commercial kit from Nanjing Jian Cheng Bioengineering Institute, Nanjing, China.

For assays of FAS, G6PD and 6PGD, cells were homogenized by sonication in extraction buffers (0.02 M Tris-HCl, 0.25 M sucrose, 2 mM EDTA, 0.1 M sodium fluoride, 0.5 mM phenyl methyl sulphonyl fluoride, and 0.01 M β-mercaptoethanol, pH7.4). FAS activity was determined by the method of Chakrabarty and Leveille [43], G6PD activity following Barroso *et al.* [44], and 6PGD activity according to the method of Hisar *et al.* [45]. For CPT I activity determination, cells were homogenized by sonication in other extraction buffers (250 mM sucrose, 1 mM EDTA, 20 mM HEPES, and 0.5% bovine serum albumin, pH7.4). The reaction mixture contained 0.1 mM 5,5′-dithiobis(2-nitrobenzoic acid) (DTNB), 5 mM l-carnitine and 0.1 mM palmitoyl-CoA. CPT I activity was measured using the method of Morash *et al.* [46], based on monitoring the initial rate of CoA-SH release with DTNB at 412 nm. The protein content was measured photometrically by using the Bradford [47] method with bovine serum albumin as standard. One unit for enzyme activity was defined as 1 µmol of substrate converted to product at the assay temperature (28 °C) per minute. All enzyme activities were expressed per milligrams of cellular soluble protein.

### 4.3. Quantitative Polymerase Chain Reaction (PCR)

The distribution and expression levels of *STAT* mRNAs were examined by the quantitative real-time PCR (Q-PCR) method. Extraction of total RNA from fish tissues and hepatocytes, and the first strand cDNA synthesis were performed as described above. Q-PCR assays were performed in a quantitative thermal cycler (MyiQ™ 2 TwoColor Quantitative PCR Detection System, BIO-RAD, Hercules, CA, USA) with a 20 µL reaction volume containing 10 µL SYBR Premix Ex Taq™ II (TaKaRa, Japan), 1 µL of diluted cDNA (10-fold), 10 mM each of forward and reverse primers 0.4 and 8.2 µL H_2_O. Primers are given in Supplementary Table S2. The Q-PCR parameters consisted of initial denaturation at 95 °C for 30 s, followed by 40 cycles at 95 °C for 5 s, 57 °C for 30 s and 72 °C for 30 s. All Q-PCR reactions were carried out in duplicate. Agarose gel electrophoresis and melting curves were performed to ensure only a single product of the correct size was amplified. And a non-template control was included to confirm that stock solutions were not contaminated. Standard curves were constructed for each target gene using serial dilutions of stock cDNA to account for any differences in efficiencies of amplification.

The amplification efficiencies of all these genes ranged between 98% and 103%. A set of eight common housekeeping genes (*β-Actin*, *18 s rRNA*, *GAPDH*, *HPRT*, *RPL7*, *TBP*, *TUBA*, and *UBCE*) were selected from the literature [48] in order to test their transcription stability. The two most stable control genes (tissues distribution: β-Actin and 18 s rRNA, *M* = 0.76; experimental conditions: *RPL7* and *TBP*, geNorm *M* value (*M*) = 0.30) were selected by using geNorm software [48]. The expression levels of each gene were normalized to the geometric mean of the best combination of two genes, which were selected by geNorm. Fold change in relative expression to the control was calculated by the “Δ*C*t” method [49].

### 4.4. Statistical Analysis

All statistical analyses were conducted with SPSS 17.0 software (SPSS, Michigan Avenue, Chicago, IL, USA). Results were presented as mean ± standard error of mean (SEM). Prior to statistical analysis, the data were evaluated for normality using the Kolmogorov-Smirnov test. The Barlett’s test was performed for testing homogeneity of variances. Then, data were subjected to one-way ANOVA followed by Turkey’s multiple range tests. Significant differences were determined at *p* < 0.05.

## 5. Conclusions

In conclusion, we cloned and characterized seven *STAT* family members from *S. hasta*. The phylogenetic analysis provided evidence that the seven *STAT* members had arisen by WGDs during vertebrate evolution. The widespread tissue expression reflected that distinct tissues were potential STAT targets in various biological roles. Leptin administration *in vitro* reflected the role in regulating the processes of hepatic lipid metabolism presumably via the STATs. Considering the subsequent change on genes expression caused by AG490 intervention, our results suggested that the lipolytic action of leptin was mainly exerted through the STAT3 member.

## Figures and Tables

**Table 1:** The sequence information of *STATs* (signal transducers and activators of transcription proteins).

Gene	Accession No.	5′ UTR (bp)	ORF (bp)	3′ UTR (bp)	Full Length (bp)	Protein (aa)
*STAT1-1*	KU532278	68	2292	915	3275	763
*STAT1-2*	KU532279	68	2253	915	3236	750
*STAT2*	KU532280	170	2484	1068	3722	827
*STAT3-1*	KU532281	224	2358	1090	3672	785
*STAT3-2*	KU532282	224	2298	1090	3612	765
*STAT4*	KU532283	146	2229	429	2804	742
*STAT5.1*	KU532284	180	2361	624	3165	786
*STAT5.2*	KU532285	125	2352	386	2863	783
*STAT6*	KU532286	462	2250	244	2956	749

**Table 2:** Amino acid sequence identity of STAT between different species (%) (Sh, Dr, Ga, Tr and Hs mean *S. hasta*, *Danio rerio*, *Gasterosteus aculeatus*, *Takifugu rubripes* and *Homo sapiens*, respectively). Amino acid sequence identity of the same STAT isoform between species is shaded. Values in the bottom line represent the means ± SEM from identity of the same STAT isoform.

Species	STAT1					STAT2					STAT3					STAT4					STAT5.1					STAT5.2				STAT6				
	Sh	Dr	Ga	Tr	Hs	Sh	Dr	Ga	Tr	Hs	Sh	Dr	Ga	Tr	Hs	Sh	Dr	Ga	Tr	Hs	Sh	Dr	Ga	Tr	Hs	Sh	Dr	Ga	Hs	Sh	Dr	Ga	Tr	Hs
Sh S1	***	61.5	80.4	80.7	67.7	42.2	43.3	44.1	44.7	39.4	50.4	49.4	50.3	51.0	50.5	48.3	51.3	49.7	48.9	49.2	27.4	27.3	27.8	27.7	27.1	26.7	27.3	26.4	27.0	24.2	25.1	24.5	23.7	23.0
Dr S1	–	***	63.3	62.5	61.9	42.7	42.5	42.4	42.7	37.6	47.3	47.4	47.8	47.7	47.9	46.7	49.8	48.7	47.7	48.7	28.0	28.2	27.9	27.7	27.6	27.4	27.0	26.7	26.7	26.0	27.0	25.2	25.0	25.2
Ga S1			***	83.8	67.6	41.3	42.5	42.9	44.4	39.7	50.3	50.0	50.5	50.9	51.1	47.4	50.1	48.6	48.8	48.7	26.7	26.6	26.9	26.8	26.7	26.1	26.2	25.8	26.4	24.0	25.4	24.4	23.9	23.1
Tr S1				***	67.7	40.9	41.5	42.4	43.6	39.3	49.7	48.8	49.3	49.9	50.0	47.9	51.2	49.0	48.8	49.5	26.3	26.5	26.7	26.7	26.6	25.5	26.2	25.6	26.0	24.8	25.2	24.0	23.3	22.0
Hs S1					***	44.2	46.4	45.3	46.1	42.3	51.4	51.2	51.6	52.2	51.8	53.1	53.6	53.3	52.3	53.3	28.0	28.0	27.9	27.8	28.6	27.4	28.3	27.0	28.4	26.8	26.7	26.2	25.3	23.7
Sh S2						***	53.6	69.7	69.6	38.3	41.7	41.4	41.3	41.9	41.4	39.0	40.9	39.9	38.9	40.4	24.3	24.2	23.9	24.1	24.1	23.1	23.5	23.1	24.3	24.2	24.4	25.0	24.2	22.3
Dr S2							***	56.9	58.5	36.3	42.7	41.7	42.1	43.0	42.9	39.8	40.9	39.7	39.2	41.5	27.1	27.0	27.3	27.4	26.2	27.2	26.7	27.2	26.4	25.0	25.3	26.1	24.3	22.6
Ga S2								***	74.5	43.1	43.2	43.0	43.2	43.5	43.2	39.5	41.1	40.1	39.1	41.1	25.3	25.4	26.0	25.9	26.5	24.6	25.4	24.9	26.3	24.9	25.4	25.8	24.6	23.2
Tr S2									***	41.9	44.3	44.6	44.3	44.6	44.1	40.9	41.7	40.7	40.9	41.8	26.8	26.6	26.8	26.5	27.0	26.4	27.0	26.6	27.0	25.7	26.2	26.4	26.3	24.8
Hs S2										***	36.9	36.2	35.8	36.8	37.5	38.5	41.0	40.3	40.0	40.3	23.8	24.4	23.3	23.3	23.9	23.9	24.0	24.8	23.2	20.7	23.5	22.5	21.6	20.6
Sh S3											***	95.7	97.1	96.3	88.7	47.5	48.6	47.9	47.1	48.5	29.1	29.5	29.5	29.3	29.7	29.3	29.2	29.5	30.0	22.7	25.9	24.9	23.8	22.0
Dr S3												***	92.3	94.5	88.5	47.4	48.9	48.1	47.5	48.7	28.5	29.2	29.1	28.8	28.8	28.9	28.7	29.1	28.9	22.5	25.0	24.9	23.5	21.2
Ga S3													***	95.3	87.9	47.9	49.0	48.0	47.3	49.2	28.4	29.1	29.0	28.7	29.2	28.5	28.4	28.9	29.3	22.7	25.2	25.1	23.2	21.6
Tr S3														***	88.0	47.8	49.1	48.2	47.4	48.6	28.8	29.3	29.3	29.1	29.8	28.8	29.2	29.3	30.1	22.6	25.4	25.1	23.6	22.3
Hs S3															***	47.0	48.8	47.9	46.8	48.2	28.2	28.6	28.7	28.6	28.6	28.3	28.9	28.7	28.8	22.4	25.2	25.0	24.0	22.6
Sh S4																***	73.2	86.0	81.7	59.1	27.0	28.0	27.7	27.5	28.4	27.7	27.9	27.9	28.5	25.8	25.7	25.4	25.9	25.8
Dr S4																	***	74.8	72.4	61.0	28.6	28.9	28.8	28.6	28.9	27.8	27.9	27.9	28.6	25.1	26.6	25.4	26.4	26.4
Ga S4																		***	86.1	59.3	28.4	29.6	29.2	29.0	29.3	28.7	28.0	28.5	29.0	24.9	26.5	25.3	25.3	25.2
Tr S4																			***	58.3	27.7	28.3	28.2	27.9	28.4	28.3	27.9	28.0	28.3	25.0	26.4	25.4	25.5	25.3
Hs S4																				***	29.4	29.8	29.5	29.2	29.8	28.8	28.9	28.7	29.5	24.8	25.6	25.3	25.9	24.1
Sh S5.1																					***	92.6	94.3	94.9	79.1	81.0	82.7	81.5	78.0	34.9	34.6	39.0	34.9	36.7
Dr S5.1																						***	94.1	93.6	80.1	82.2	83.9	82.2	79.1	35.9	35.0	39.1	35.5	37.9
Ga S5.1																							***	96.3	79.6	82.3	82.8	82.0	78.2	35.3	34.4	39.4	35.2	36.9
Tr S5.1																								***	79.9	82.7	83.2	82.5	78.7	35.5	34.8	39.5	35.5	37.7
Hs S5B																									***	74.2	74.2	74.8	93.7	34.6	33.9	37.9	35.4	37.8
Sh S5.2																										***	79.5	89.6	73.6	35.6	35.5	39.0	35.2	37.2
Dr S5.2																											***	78.6	74.5	35.5	35.6	38.6	35.1	37.0
Ga S5.2																												***	74.0	34.2	34.0	38.0	35.5	37.0
Hs S5A																													***	34.6	34.1	38.2	35.2	37.7
Sh S6																														***	50.9	58.3	57.6	38.3
Dr S6																															***	58.7	53.7	37.3
Ga S6																																***	74.4	41.4
Tr S6																																	***	37.4
Hs S6																																		***
Values	69.71 ± 2.72					54.24 ± 4.43					92.43 ± 1.20					71.19 ± 3.54					88.45 ± 2.41					78.30 ± 2.48				50.80 ± 3.85				

“***” represents the amino acid sequence identity of the same STAT.

## Supplementary Materials

Supplementary materials can be found at http://www.mdpi.com/1422-0067/17/3/406/s1.

## Abbreviations

6PGD: 6-phosphogluconate dehydrogenase
CPT I: carnitine palmitoyl transferase I
FAS: fatty acid synthase
G6PD: glucose-6-phosphate dehydrogenase
GAPDH: glyceraldehyde-3-phosphate dehydrogenase
HPRT: hypoxanthine-guanine phosphoribosyl transferase
JAK: janus kinase
LepR: leptin receptor
MS-222: tricaine methanesulfonate
RPL7: ribosomal protein L7
STAT: signal transducer and activator of transcription
TBP: TATA-box-binding protein
TG: triglyceride
TUBA: htubulin alpha chain
UBCE: ubiquitin-conjugating enzyme
WGB: whole genome duplication
