# Supplementary material for: Isolation and Expression Analysis of STAT Members from Synechogobius hasta and Their Roles in Leptin Affecting Lipid Metabolism
Source: Int J Mol Sci. 2016 Mar 22;17(3):406. doi: 10.3390/ijms17030406 (PMC4813261; doi:10.3390/ijms17030406)
Supplement: Supplementary file 1 [file ijms-17-00406-s001.pdf]

Supplementary Materials: Isolation and Expression Analysis of STAT Members from Synechogobius hasta and Their Roles in Leptin Affecting Lipid Metabolism

Kun Wu, Xiao-Ying Tan, Chuan-Chuan Wei, Wen-Jing You, Mei-Qin Zhuo and Yu-Feng Song

Table S1. Protein ID numbers of target sequences in different species.

Species	Isoform	Protein ID
Homo sapiens	STAT1	ENST00000361099
	STAT2	ENST00000314128
	STAT3	ENST00000264657
	STAT3β	ENST00000585517
	STAT4	ENST00000392320
	STAT5A	ENST00000345506
	STAT5B	ENST00000293328
	STAT6	ENST00000300134
Rattus norvegicus	STAT1	ENSRNOT00000019465
	STAT2	ENSRNOT00000049536
	STAT3	ENSRNOT00000026760
	STAT4	ENSRNOT00000020032
	STAT5A	ENSRNOT00000026662
	STAT5B	ENSRNOT00000026354
	STAT6	ENSRNOT00000039306
Xenopus tropicalis	JAK1	ENSXETT00000044013
	JAK2	ENSXETT00000001294
	JAK3	ENSXETT00000035893
	TYK2	ENSXETT00000025411
	STAT1	ENSXETT00000010105
	STAT2	ENSXETT00000047227
	STAT4	ENSXETT00000010124
	STAT5B	ENSXETT00000001864
	STAT6	ENSXETT00000009652
Latimeria chalumnae	STAT3	ENSLACT00000013753
Ciona intestinalis	STAT-A	ENSCINT00000020503
	STAT-B	ENSCINT00000008341
Caenorhabditis elegans	STAT	Y51H4A.17a
Danio rerio	STAT1a	ENSDART00000005720
	STAT1b	ENSDART00000145674
	STAT2	ENSDART00000148661
	STAT3	ENSDART00000104519
	STAT4	ENSDART00000105764
	STAT5.1	ENSDART00000024832
	STAT5.2	ENSDART00000124638
	STAT6	ENSDART00000023218

Table S1. *Cont.*

Species	Isoform	Protein ID
Oryzias latipes	STAT1	ENSORLT00000000200
	STAT2	ENSORLT00000002051
	STAT3	ENSORLT00000005086
	STAT4	ENSORLT00000019022
Oreochromis niloticus	STAT1	ENSONIT00000015373
	STAT2	ENSONIT00000024057
	STAT3	ENSONIT00000001452
	STAT4	ENSONIT00000021209
	STAT5.1	ENSONIT00000001431
	STAT5.2	ENSONIT00000009161
	STAT6	ENSONIT00000015034
Takifugu rubripes	STAT1	ENSTRUT00000000223
	STAT2	ENSTRUT00000033362
	STAT3	ENSTRUT00000039373
	STAT4	ENSTRUT00000028160
	STAT5.1	ENSTRUT00000039672
	STAT6	ENSTRUT00000040273
Gasterosteus aculeatus	STAT1	ENSGACT00000020606
	STAT2	ENSGACT00000000913
	STAT3	ENSGACT00000011411
	STAT4	ENSGACT00000003538
	STAT5.1	ENSGACT00000011424
	STAT5.2	ENSGACT00000020355
	STAT6	ENSGACT00000011232

Table S2. Primers used for cDNA cloning and Q-PCR of genes from *S. hasta*.

Gene	Forward Primer (5′–3′)	Reverse Primer (5′–3′)	Step	Size	Accession No.
STAT1	GTHATGAACATGGARGAGTCC	AGWCGTCBGGDGACATBGGCAT	Partial fragment	1016	KU532278
	TTCCTSMWVCARCACAACATC	GCCTCCAGTCGGATGTCCAG	Partial fragment	1109	
	GCCATACACGAAGAAGGAGC	TACCGTCGTTCCACTAGTGATTT	3′ RACE Outer	1321	
	CTGAGAAGAGTGGCTACATGA	CGCGGATCCTCCACTAGTGATTTCACTATAGG	3′ RACE Inner	1130	
	CATGGCTACATGCTGACAGCCTA	CTTTGCCACCACGTCCTGCC	5′ RACE Outer	710	
	CGCGGATCCACAGCCTACTGATGATCAGTCGATG	TCCCCCTCTTGCTCTGTACT	5′ RACE Inner	448	
	AGCTCAACATGCTGGCCGAC	TCCATAGCCAGAACGGGAAC	Q-PCR	126	
STAT2	GGARGAGCAGCARGAYGAGTT	CAGGCRCTYTGCTGCTGRCTGGAGTT	Partial fragment	897	KU532280
	CAGAAGTGCGGAGGCGGAG	CAYCAGCKTRSTTYTGATGTA	Partial fragment	861	
	TGGGTCGAAACTACGCTCAC	TACCGTCGTTCCACTAGTGATTT	3′ RACE Outer	1728	
	ATCCAAACACGCCCAAAGAG	CGCGGATCCTCCACTAGTGATTTCACTATAGG	3′ RACE Inner	1574	
	CATGGCTACATGCTGACAGCCTA	GAGCTCCGATGCAGGACTTC	5′ RACE Outer	915	
	CGCGGATCCACAGCCTACTGATGATCAGTCGATG	CTGATTCACAAGAGACTGGAGG	5′ RACE Inner	779	
	ATGCTCAGCCTCGACACCA	TTGCTCTTCATTCAATCCGACT	Q-PCR	129	
STAT3	CAGCAYCTRCARAGCAAGTA	GTTVGAYTCYTCCATGTTCAT	Partial fragment	942	KU532281
	GGTGAAGTTTCCAGAGCTGA	TSDGCBTCRKTGTGCATCAG	Partial fragment	1239	
	CCGACATCATCATGGGCTAC	TACCGTCGTTCCACTAGTGATTT	3′ RACE Outer	1494	
	GCCGTATTTGAAGACCAAGTTC	CGCGGATCCTCCACTAGTGATTTCACTATAGG	3′ RACE Inner	1325	
	CATGGCTACATGCTGACAGCCTA	AGTTGAGCCATTTTCTGTCGC	5′ RACE Outer	835	
	CGCGGATCCACAGCCTACTGATGATCAGTCGATG	CTTCTCGGTGACCACTGTCC	5′ RACE Inner	641	
	ACATGCTAACAAACCACCCGA	ACTGCCAGCTCAGGACCTCA	Q-PCR	93	
STAT3-1	GAGCCAGACATGGTAGGAGG	AGATCGCTGTCCGGTAGGTC	Q-PCR	116	KU532281
STAT3-2	TGCGTTACCCCCACAAATTC	TCGTTGTGCATGAGCGAGT	Q-PCR	83	KU532282
STAT4	ACAARACNCTHCAGTCTCGAG	TTRTAGTCYCGRATKATGTC	Partial fragment	1417	KU532283
	GACTTTCAGGTGTCCTGGTC	TACCGTCGTTCCACTAGTGATTT	3′ RACE Outer	1077	
	AGCCCGGGACTTTCCTCCT	CGCGGATCCTCCACTAGTGATTTCACTATAGG	3′ RACE Inner	893	
	CATGGCTACATGCTGACAGCCTA	GTTCTGCAGTTGTTCCAGGC	5′ RACE Outer	917	
	CGCGGATCCACAGCCTACTGATGATCAGTCGATG	CTATGTCCATCTTAGACAGG	5′ RACE Inner	792	
	TGGTCATCTCCAACGTGAGC	CAACTCAGAACCTCCGACAGC	Q-PCR	145	

Table S2. *Cont.*

Gene	Forward Primer (5′–3′)	Reverse Primer (5′–3′)	Step	Size	Accession No.
STAT5.1	GACCGVCAGCTMMGRGAGCC	TRGMRAAVACCTCATCYTTGG	Partial fragment	1468	KU532284
	AACAGCACTGGAACGATGGT	TACCGTCGTTCCACTAGTGATTT	3′ RACE Outer	1229	
	CAGGGGAGAGGATGGTGTGG	CGCGGATCCTCCACTAGTGATTTCACTATAGG	3′ RACE Inner	1070	
	CATGGCTACATGCTGACAGCCTA	TCTCTGCCAACTTCTCACAC	5′ RACE Outer	1033	
	CGCGGATCCACAGCCTACTGATGATCAGTCGATG	AGCTGCAGGGTTTTCTGGTG	5′ RACE Inner	896	
	GAAAGCAGCGGGGACATTC	ACTCCGCTCCTCGTCGAT	Q-PCR	130	
STAT5.2	CARCACDYTRCAGAARTACAG	ARRTGGTTCAGRTCACTGAT	Partial fragment	1353	KU532285
	GCAATGGTTTGAGGGAGTTGT	TACCGTCGTTCCACTAGTGATTT	3′ RACE Outer	1023	
	AGATTGGCGGCATCACCATC	CGCGGATCCTCCACTAGTGATTTCACTATAGG	3′ RACE Inner	868	
	CATGGCTACATGCTGACAGCCTA	GGCAGAAATGATGTCCGTGA	5′ RACE Outer	1106	
	CGCGGATCCACAGCCTACTGATGATCAGTCGATG	CCAGTTTCTCACACCAGGAC	5′ RACE Inner	975	
	TCGGGTGCCTTTCATTGTC	TGGGTTATTACTGGTGCTGCTA	Q-PCR	160	
STAT6	MRGATGAAGCTSDTGCAGAT	GTYAGMTCCATBRCTCCCTC	Partial fragment	1400	KU532286
	TCAATGCTATGGCCACAGTTC	TACCGTCGTTCCACTAGTGATTT	3′ RACE Outer	1161	
	CTACAACCTGCTGTTTCTGGC	CGCGGATCCTCCACTAGTGATTTCACTATAGG	3′ RACE Inner	1007	
	CATGGCTACATGCTGACAGCCTA	CAGACCCTTGTGGTGCCATT	5′ RACE Outer	1000	
	CGCGGATCCACAGCCTACTGATGATCAGTCGATG	GTGATCTCTGGAGCTGTGGG	5′ RACE Inner	878	
	CACAAAGCTGCCATCGGACA	ACCAAATACACCTCCTGACGC	Q-PCR	110	
LepR	CCTCAAAACAGCCAAGCACC	CGCTCTGAGGCTGGTATCTG	Q-PCR	166	KU359049
CPT I	CGCTCCTGCTCCAATGAGA	GAGACCACATAGAGGCAGAAGA	Q-PCR	173	KJ508197
6PGD	CTGCTGCTGGACTCCTTCTT	GTTGTGTCTGTAACCGTCGTAA	Q-PCR	144	KJ508184
G6PD	GAGAAGCCTGCCTCAACCA	GGATCGTCCAAGTAGCCAAGT	Q-PCR	158	KJ508185
FAS	CATCATCACTGGAGGTCTTGGA	TACGAATGCCTGATCTGGAAGT	Q-PCR	104	KJ508188
β-Actin	GTGCGTGACATCAAGGAGAAG	CGAGGAAGGATGGCTGGAA	Q-PCR	178	KP893561
18s rRNA	TCATTCCGATAACGAACGAG	GGACATCTAAGGGCATCACA	Q-PCR	143	KP893562
GAPDH	AAAGTCATCCCCGAGCTCAA	CTTCAGACGCAGCCTTCATC	Q-PCR	148	KP893555
HPRT	CCTCTCCGACTCACAGCTAG	GTCGCCATCTTCACCTCAAC	Q-PCR	136	KP893556
RPL7	GCGCCAGATCTTCAATGGAG	CTCATTCTGCCATGACCACG	Q-PCR	147	KP893557
TBP	AGTCCCATGATGCCCTATGG	GCAACAGCTTGGGAATGGAA	Q-PCR	179	KP893559
TUBA	CACTTCCCTCTTGCCACCTA	ACGGTACAGGAGACAACAGG	Q-PCR	165	KP893558
UBCE	GCCCGTGGAAGGATTCAAAA	AAGGCAGGTGGAGAGTATGG	Q-PCR	156	KP893560
